# Editorial: Advanced neurotechnology in stroke rehabilitation

**DOI:** 10.3389/fneur.2024.1440752

**Published:** 2024-06-20

**Authors:** Jingyao Sun, Chong Li

**Affiliations:** School of Clinical Medicine, Tsinghua University, Beijing, China

**Keywords:** stroke rehabilitation, advanced neurotechnology, brain machine interface (BMI), technology-aided neurorehabilitation, rehabilitation mechanism

Stroke is the leading cause of death and disability worldwide, impacting patients and their families economically (1). Motor impairment emerges as the most common consequence of stroke; more than 80% of stroke survivors have acute impairment, and more than 50% have chronic impairment (2). Currently, advanced neurotechnologies, including but not limited to rehabilitation robotics (3), brain machine interaction (BMI) (4, 5), brain stimulation (6), functional electrical stimulation (FES) (7), aimed at restoring motor function have attracted huge attention from professionals and public. Recent clinical studies on these approaches have shown promising results (8). However, there remains a need for more extensive studies with larger sample sizes to unravel the mechanisms underlying technology-aided neurorehabilitation and to maximize rehabilitative effects. Therefore, this Research Topic aimed to investigate recent clinical applications of these neurotechnologies, in order to better identify current research or clinical gaps, optimize existing techniques and maximize the rehabilitative outcomes.

This Research Topic collection comprised four articles, including two original studies and two review studies. The discussed research delved into the underlying recovery mechanisms after stroke and clinical applications in facilitating motor recovery.

In the study by Park et al., the authors aimed to assess the correlation of temporal muscle thickness (TMT) with grip strength to develop a new biomarker for predicting patient's sarcopenia. The results indicated that TMT was associated with grip strength and sarcopenia risk in hemiplegic patients, providing a basis for predicting a patient's pre-stroke muscle strength status. In the study by Jiang et al., the authors paid attention to post-stroke urinary incontinence (PSUI), which is characterized by urinary frequency, urgency, and uncontrolled urine flow from the urethra. The researchers utilized meta-analysis and assessed the efficacy of electroacupuncture (EA) for PSUI. The meta-analysis results suggested that EA improved post-stroke urinary incontinence with no serious adverse effect.

Furthermore, Xie et al. assessed the therapeutic effects of walking training assisted with soft robotic exoskeleton (SRE) on clinical and biomechanical gait outcomes in the rehabilitation of patients with subacute stroke. The results demonstrated that SRE-assisted walking training achieved greater improvements in walking speed, endurance, and motor recovery, compared with conventional rehabilitation training. The study highlights the potential benefits of SRE-assisted rehabilitation techniques and provides preliminary evidence that SRE may be considered for inclusion in intensive gait training clinical rehabilitation programs. In the study by Okamura et al., the authors conducted a meta-analysis to evaluate the effects of virtual reality-based mirror therapy (VRMT) on upper extremity dysfunction in stroke survivors. The meta-analysis included five randomized controlled trials with 148 stroke patients and indicated statistical differences in the results of Fugl–Meyer assessment upper extremity test (FMA-UE) between the VRMT and the control groups. The authors concluded that VRMT might play a beneficial role in improving upper extremity dysfunction after stroke, especially when combined with conventional rehabilitation.

Taken together, the collective insights gained from the studies above have significantly enriched our understanding of technology-aided neurorehabilitation. However, further steps are required for the clinical implementation of these neurotechnologies. This gap highlights the need for a comprehensive understanding of the underlying mechanisms through which these approaches influence functional recovery in both preclinical and clinical settings. Additionally, it also emphasizes the importance of developing biomarkers capable of stratifying patients and predicting their responsiveness to different types of neurotechnologies. Further studies on larger samples are needed, based on careful sample selection, rigorous methodology, and follow-up to increase the reliability and promote clinical translation of advanced neurotechnologies.

## Author contributions

JS: Writing – original draft. CL: Writing – original draft, Writing – review & editing.

## References

[1] GBD 2019 Stroke Collaborators. Global, regional, and national burden of stroke and its risk factors, 1990-2019: a systematic analysis for the Global Burden of Disease Study 2019. Lancet Neurol. (2021) 20:795-820. 10.1016/S1474-4422(21)00252-0 34487721 PMC8443449

[2] Micera S Caleo M Hummel FC Pedrocchi A. Advanced neurotechnologies for the restoration of motor function. Neuron. (2020) 105:604–20. 10.1016/j.neuron.2020.01.039 32078796

[3] Rodgers H Bosomworth H Krebs HI van Wijck F Howel D Wilson N. Robot assisted training for the upper limb after stroke (RATULS): a multicentre randomised controlled trial. Lancet. (2019) 394:51–62 10.1016/S0140-6736(19)31055-4 31128926 PMC6620612

[4] Khademi F Naros G Nicksirat A Kraus D Gharabaghi A. Rewiring cortico-muscular control in the healthy and post-stroke human brain with proprioceptive beta-band neurofeedback. J Neurosci. (2022) 42:6861–77. 10.1523/JNEUROSCI.1530-20.2022 35940874 PMC9463986

[5] Sun J Jia T Li Z Li C Ji L. Enhancement of EEG-EMG coupling detection using corticomuscular coherence with spatial-temporal optimization J Neural Eng (2023) 20 10.1088/1741-2552/accd9b 37068482

[6] Baker KB Plow EB Nagel S Rosenfeldt AB Gopalakrishnan R Clark C. Cerebellar deep brain stimulation for chronic post-stroke motor rehabilitation: a phase I trial. Nat Med. (2023) 29:2366–74. 10.1038/s41591-023-02507-0 37580534 PMC10504081

[7] Biasiucci A Leeb R Iturrate I Perdikis S Al-Khodairy A Corbet T. Brain-actuated functional electrical stimulation elicits lasting arm motor recovery after stroke. Nat Commun. (2018) 9:2421. 10.1038/s41467-018-04673-z 29925890 PMC6010454

[8] Ganguly K Khanna P Morecraft RJ Lin DJ. Modulation of neural co-firing to enhance network transmission and improve motor function after stroke. Neuron. (2022) 110:2363–85 10.1016/j.neuron.2022.06.024 35926452 PMC9366919

